# Evaluation of factors associated with fear and anxiety in the orthodontic treatment of adult patients

**DOI:** 10.4317/jced.62265

**Published:** 2025-01-01

**Authors:** Lucía Serrat-Lacasta, Susana de la Cruz-Vigo, Manuele Leonelli

**Affiliations:** 1 Clínica Universitaria Odontológica de la Universidad Europea de Madrid. Paseo Santa Maria de la Cabeza 92.28045 Madrid. Spain

## Abstract

**Background:**

Despite the technological advances made in dentistry, anxiety and fear of pain due to dental treatment are still common. Most patients in a dental clinic suffer some symptoms of anxiety, which can appear at different times. The objective is to know the degree of anxiety and the factors associated with this anxiety during orthodontic treatment.

**Material and Methods:**

180 adult patients who started orthodontic treatment were selected and a questionnaire was administered at the beginning and after six months of treatment. The SDAI anxiety scale was used to determine the degree of anxiety and its own questionnaire to determine its relationship with the different factors of orthodontic treatment.

**Results:**

When performing descriptive and inferential statistics, it is obtained that 72% of patients at the beginning of orthodontic treatment present a certain degree of anxiety. Through individual logistic regression using anxiety as a response and each of the demographic variables at six months as predictors, it is obtained that gender(OR=3.15) and occupation(OR=3.11) are the two significant predictors (*P*<0, 05). Using Fisher’s exact test of independence, the additional questions are related to the demographic variables and anxiety, obtaining a value of *P*<0.05 in 27(of 114) non-independent relationships between the additional questions and the demographic/anxiety variables.

**Conclusions:**

38%of patients who begin orthodontic treatment have high anxiety, but after 6 months this anxiety drops to 22%. Women and patients with active work are the most likely to present more anxiety at 6 months. The biggest concerns at the beginning of treatment are: Changes in appearance(Q15),Eating(Q18),Appliance loss(Q19),Hygiene(Q24),Tooth loss(Q25),Extend treatment(Q26),Expectations(Q27) and Retention(Q28).Of these eight, only three continue to worry patients after six months: appliances loss(Q19),Hygiene(Q24) and Tooth Loss(Q25).At the beginning of treatment there are two questions that do not worry: Enter alone(Q10) and take medications(Q21).Four more are added after six months: Embarrassment(Q16),Getting used to it(Q17),Periodic visits(Q22) and Emergencies(Q23).

** Key words:**SDAI scale, Dental anxiety, Adults, Orthodontic treatment.

## Introduction

Despite technical and scientific progress in dentistry, improvements in dental practices, the reduction of pain in interventions, improved hygiene habits and less invasive techniques, fear of the dentist continues to persist (1).

The visit to the dentist is one of the situations that generates most anxiety in people, occupying fifth place among the situations most feared by people (1,2), leading to people stopping going to the dental clinic and abandoning treatment. These episodes are known as dental anxiety (2). Not all procedures or stages of dental treatment produce the same level of anxiety and it can even appear in advance, just by thinking about the encounter and that an aversive experience is approaching, causing high anxiety (3).

In general, the prevalence of high dental anxiety is considered to range from 4% -20% (1,3). However, multiple studies show that 85-90% of patients experience fear or anxiety before or during dental treatment and 6-15% avoid dental care (1). Therefore, the dentist should be aware that dental treatments provoke a certain degree of anxiety, and should be prepared to identify and treat such patterns, taking concrete measures to avoid or reduce anxiety in order to improve the patient’s oral quality of life (3).

## Material and Methods

This study complies with the Declaration of Helsinki(4) and was approved by the ethics committee of the European University of Madrid (Registration 22.186). It is an observational, cross-sectional, qualitative, analytical and comparative study that was carried out in adult patients aged 18-70 years, who started orthodontic treatment with aligners and with multibracket fixed appliances in the Master’s Degree in Orthodontics at the European University Clinic of Madrid and in three private clinics in Madrid between 2022-2024. Patients were selected using a non-probabilistic method of convenience who came for orthodontic treatment. Inclusion criteria were: patients seen in these clinics; at the start of treatment with fixed appliance multibrackets or clear aligners; both sexes; voluntary participation; with or without previous experience of orthodontic treatment; fluent in Spanish; 18-65 years; with signed informed consent. Exclusion criteria were: with physical or mental disability; partial responses to the questionnaire; double answers in single responses; abandonment of treatment before 6 months from the start of treatment.

**Figure 1:**
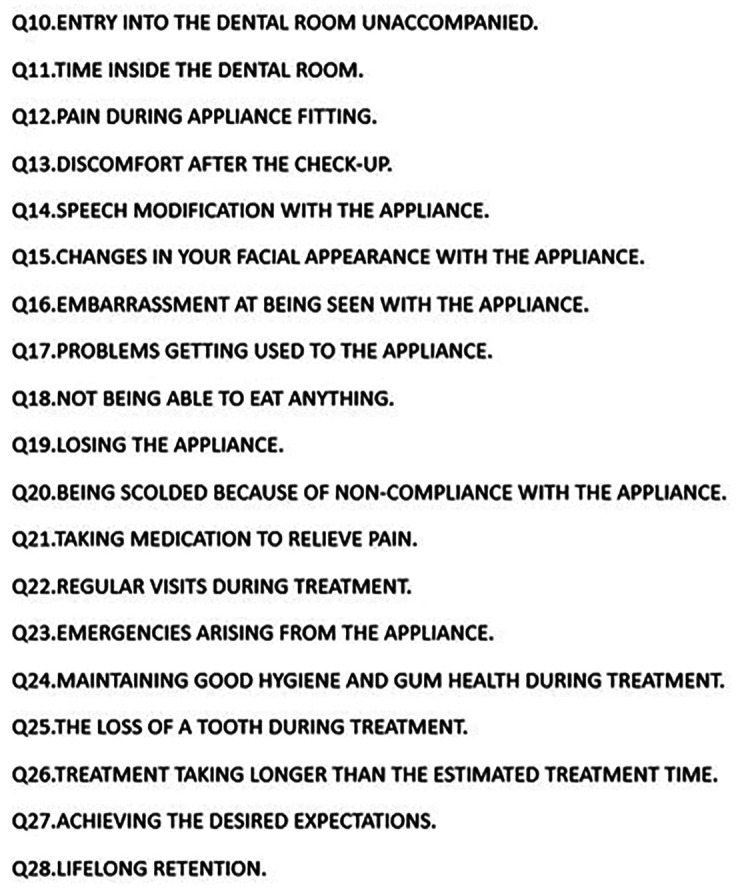
Items of our questionnaire.

Survey methodology was used, using the short version of the SDAI (Scale Dental Anxiety Index) ( Table 1), a self-report instrument developed by Stouthard M, Goen and Mellenbergh 1995. It consists of nine Lickert-type items; the response ranges were from 1 to 5: never (1 point), seldom (2 points), sometimes (3 points), very often (4 points) and always (5 points) with a Cronbach’s alpha reliability coefficient of 0.957 in its original version. The scores obtained are between 0 and 45 points: Not anxious (0 to 10 points), mildly anxious (11-19 points), moderately anxious (20-27 points), extremely anxious (28 to 45 points). The questionnaires included age, gender, level of schooling, occupation, previous treatment or not, and type of treatment; variables that were analyzed with anxiety. And a second questionnaire of our own (Fig. 1), validated by a group of orthodontists exclusively, with 19 questions, following the criteria of the OMS and Likert scale, in which the subject is given a question and asked to rate it as not at all, a little, normal, normal, a lot and too much according to their degree of agreement with it. The codes of ethics and patient care of the clinic of the Faculty of Dentistry of the University and private clinics where the study was carried out were followed and respected. They were given all the necessary information about their treatment and their participation in the study.

Statistical analysis was performed with the R program and its integrated development environment RStudio. Qualitative variables were expressed as percentages (%) and visualized using bar charts generated with the ggplot2 library. Variables were compared using Fisher’s exact test, with a level of statistical significance set at *p* ≤ 0.05. Univariate analysis using logistic regression models (for binary variables) was used to identify independent risk factors. In logistic regressions, the estimated OR (odds ratio), 95% confidence interval, and associated *p-value* are presented.

## Results

**Figure 2:**
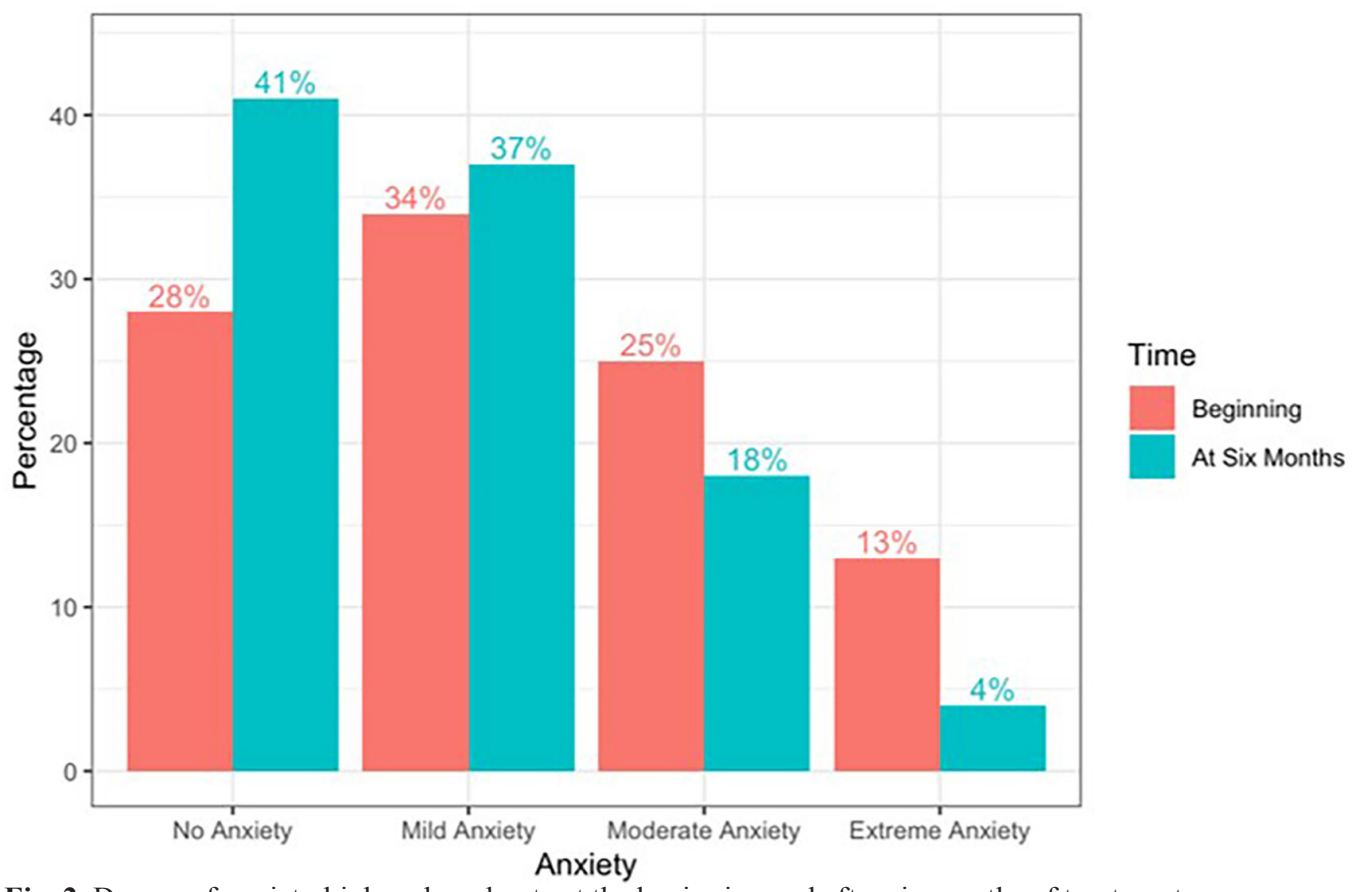
Degree of anxiety high and moderate at the beginning and after six months of treatment.

With a total of 180 patients, the degree of anxiety at the beginning of the treatment comprises: 28% without anxiety and 72% with anxiety, distributed in 34% mild anxiety, 25% moderate anxiety and 13% extreme anxiety, (Fig. 2).

After six months, anxiety decreased, leaving the following levels: 41% without anxiety, 37% mild anxiety, 18% moderate anxiety and 4% extreme anxiety (Fig. 2).

We can see that at the start of treatment no demographic variable is significantly associated with anxiety in terms of *p-value* (> 0.05). However, in terms of the OR, it can be seen that female sex, patients with a university education, active workers and patients without previous treatment have greater anxiety (odds ratio > 1) but without statistically significant differences. It is also observed that patients aged 25-44 and treated with braces have less anxiety due to their odds ratio of less than one, but the difference is not significant ( Table 2).

At 6 months of treatment two demographic variables are statistically significant, gender (odds ratio 3.148, *p-value* 0.0161): women are significantly more likely to have high levels of anxiety than men, and occupation (odds ratio 3.11, *p-value* 0.0429), as working patients are significantly more likely to have high levels of anxiety than non-working patients. The other variables are not assessable ( Table 2).

At the start of treatment there are eight questions that patients are most concerned: Changes in appearance(Q15), Eating(Q18), Appliance Loss(Q19), Hygiene(Q24), Tooth loss(Q25), Lengthening of treatment time(Q26), Expectations(Q27) and Retention(Q28). Of these eight, only three are still of concern to patients six months: Appliance loss(Q19), Hygiene(Q24) and Tooth loss(Q25).

At the start of treatment, there are two questions that patients are not concerned: Enter alone in dental room(Q10) and Take Medications(Q21). Four more are added to these two after six months of treatment: Embarrassment(Q16), Habit(Q17), Visits(Q22) and Emergencies(Q23).

We also studied how much the percentages of responses change before and after treatment ( Table 3).

When relating demographic data, additional questions and anxiety, 27 significant associations were found between anxiety and additional questions (14 before treatment and 13 at six months), ( Table 4).

Patients with high anxiety have many significant concerns both at the beginning and at 6 months. At the beginning are: (Q10, Q11, Q12, Q13, Q14, Q15, Q16, Q17, Q19, Q20, Q23, Q26, Q27, Q28). At 6 months some disappear, some are maintained and new ones appear (Q10, Q12, Q13, Q14, Q15, Q16, Q18, Q20, Q21, Q26 and Q27. In terms of age, there is only one significant question at 6 months is: the periodic visits (Q22), with less concern for the 25-44 age group than for the other age groups. In terms of education, it is seen at the beginning that university students are very concerned about getting used to the device(Q17). For non-university students, however, their main concerns at the beginning are losing the device(Q19) and achieving their expectations (Q27). At six months this group is more concerned about hygiene(Q24). In terms of occupation, there are two major significant concerns for active people at baseline; these are medication (Q21) and visits (Q22). At 6 months, there are no differences between the two groups. In terms of gender there are significant differences only at 6 months where men are more concerned about food (Q18).

Patients who have had previous treatment are significantly less concerned about the pain they may feel at the fitting (Q12), discomfort during the visit (Q13) and food (Q18).

Patients with multibracket appliances are significantly more concerned at the beginning about Food(Q18), Emergencies(Q23) and Hygiene(Q24) and at six months about Speech(Q14), Visits(Q22) and Food(Q18). In contrast, patients with aligners are more concerned only at six months about appliance loss(Q19).

## Discussion

**Figure 3:**
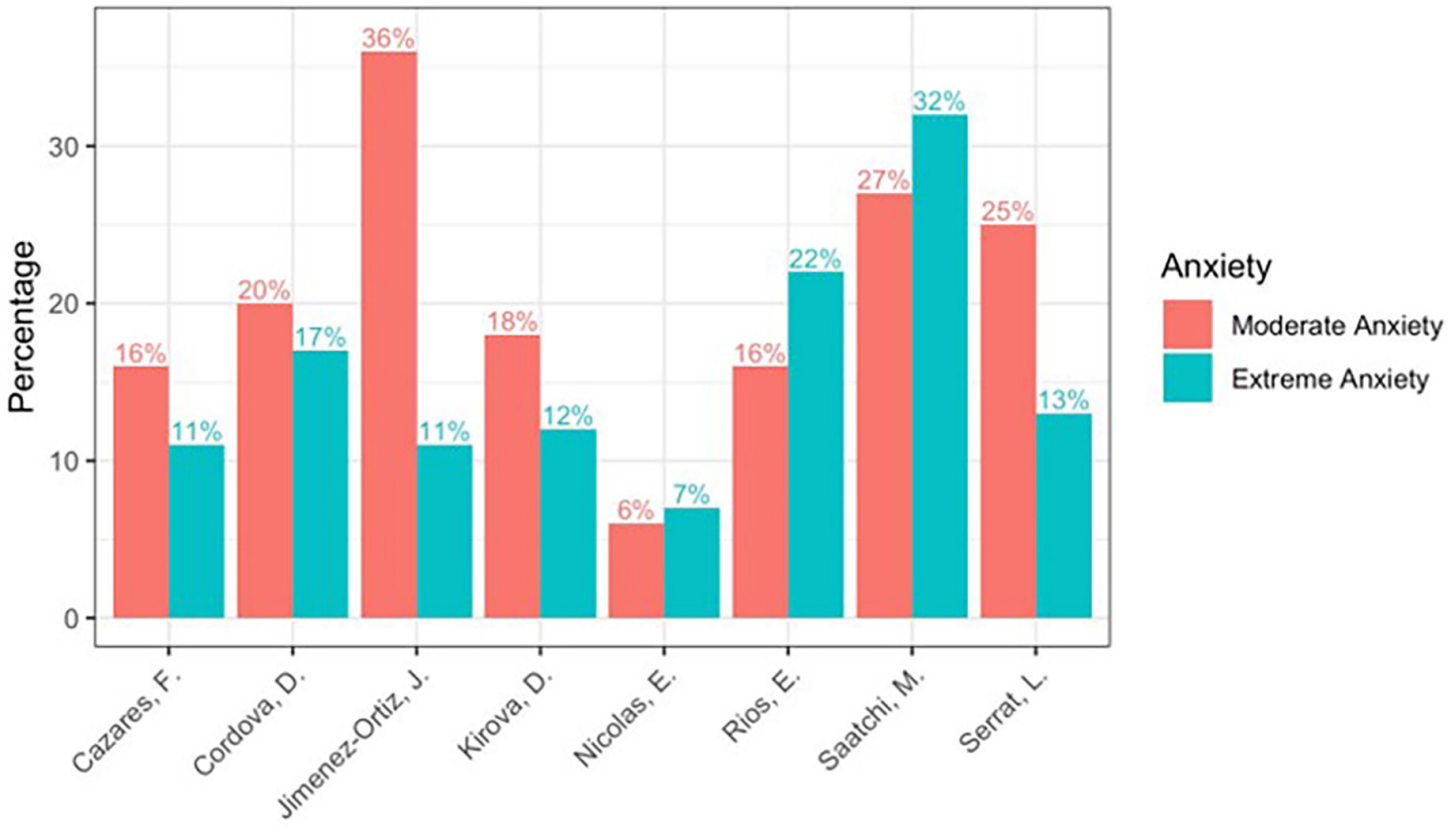
Comparison of high and moderate anxiety levels in different studies.

**Figure 4:**
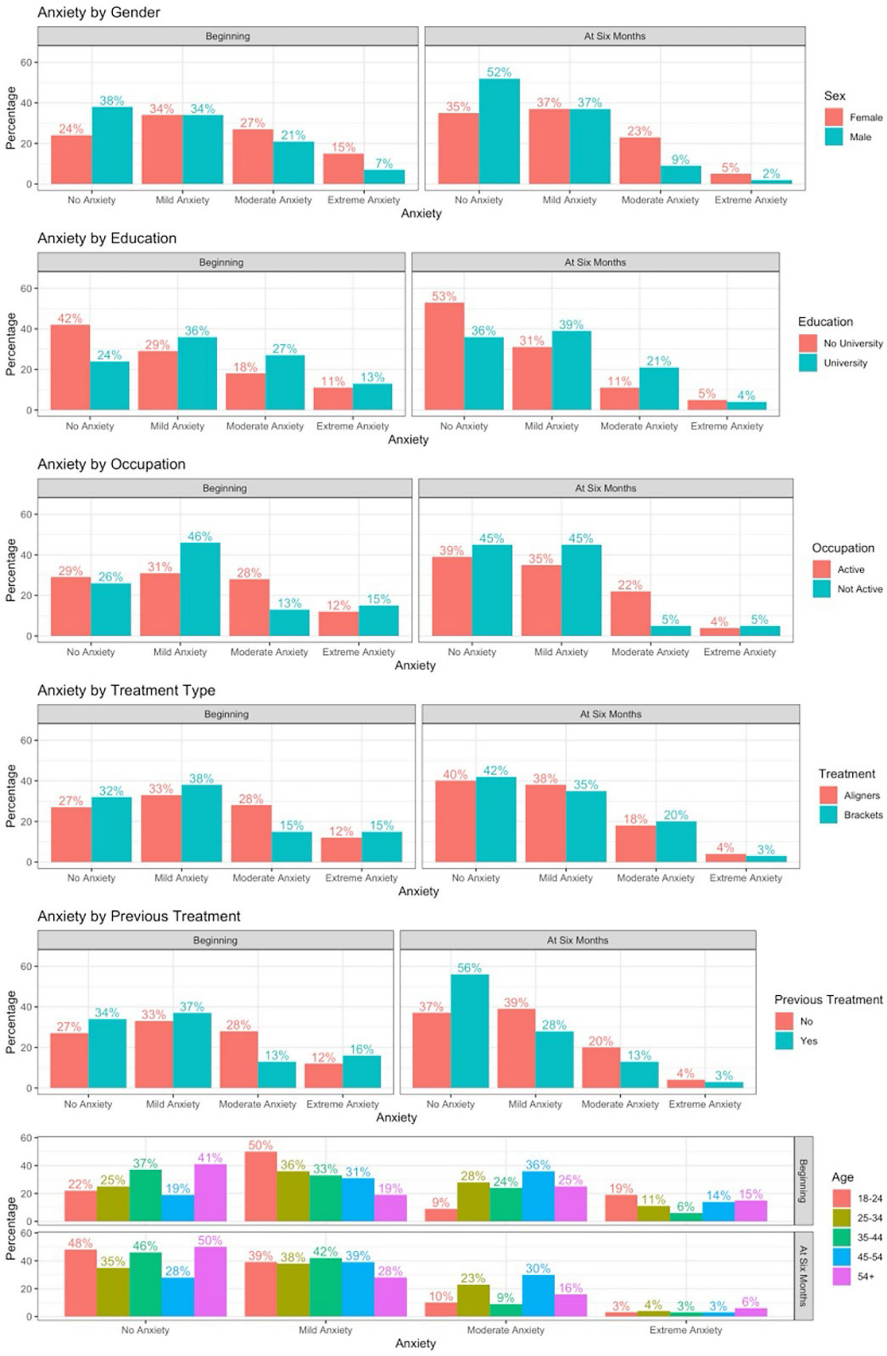
Relationship of anxiety with demographic variables at the beginning and six months of treatment.

As mentioned above, the prevalence of dental anxiety is variable according to different authors, and has varied over the years. This is probably due to the different population types and sample sizes. In the present study we obtained 38% high anxiety (moderate and severe) at the beginning of treatment, data very similar to those obtained by Córdova (5) 36.7%, and Ríos (6) 37.9%. However, there are studies that have obtained data below those of this study, such as Cazares (7) 27.1%, Nicolas (8) 13.5%, Kirova (9) 29.9%. And above our data such as Jimenez (10) 46.77%, Saatchi (11) 58.8%, (Fig. 3). In our study (Fig. 4), there is no statistically significant association between anxiety and gender, but it is observed that women are more anxious, results similar to those reported by Cazares (7), Kirova (9), Rios (6), Jimenez (10). But there are studies where statistically significant women are more anxious Saatchi (11), Caltabiano (12), Enkling (13), Appukuttan (14). There is only one article Córdova (5) that determines that men are more anxious statistically significant. Most studies report that females appear to experience greater anxiety and are more susceptible to pain in dental treatment than males. Among the causes of these differences could be considered the higher percentage of women responding to surveys and the general social acceptance of norms that allow women to express their anxieties more freely, based on different social roles and expectations and men refuse to report symptoms they consider weak or unmanly and tend to cope with anxiety in silence (15). No significant differences by age group were identified, however, in our study (Fig. 4), there is a tendency for adults between 45-55 years to present higher levels of dental anxiety than other age ranges, which coincides with the findings of Armfield (16) 40 to 64 years; Sanikop (15) over 45 years; Rios (6) even speaks of higher ages ranging from 60 to 70 years. But the vast majority of articles speak of an inversely proportional relationship between anxiety and age, i.e. as age increases anxiety decreases, finding the highest levels in young patients such as Caltabiano (12) and Appukuttan (14) aged <30 years, Enkling (13) aged 20-30 years. In our study the group with the highest extreme anxiety is 18-24 years old. This could be due to increased exposures over time, which allows patients to develop tolerance to treatment and therefore have less anxiety as they get older, as they have the ability to cope with the situation with experiences or due to the ageing process characterized by a general decrease in anxiety. But it is surprising as nowadays 20–30-year-olds should have had fewer problems in their experiences during dental treatment than older people due to the introduction of individual and group preventive measures (13). These results highlight the important heterogeneity and it is advisable to increase the sample size to check if these trends show statistical differences. Our results showed that there is no statistically significant dependence between schooling and anxiety, as in numerous studies by Cazares (7), Jimenez-Jimenez (10), Ríos (6), Enkling (13), Armsfield (16), Saatchi (11). However, in our study (Fig. 4), university patients present greater anxiety, with the same results being obtained by Cazares (7) and Jimenez-Jimenez (10). Although the vast majority of studies show an inverse relationship between anxiety and educational level, with greater anxiety with a low educational level, a fact obtained by other Ríos-Erazo (6), Appukutan (14), Kirova (9). They argue that patients with a high level of education cope better and reason better with a situation rather than avoid it and person with low educational level is among the main reasons for not seeking regular dental care and generating dental fear. In our study (Fig. 4), there is no significant association between anxiety and occupation as in several studies Cazares (7). But there are some studies that do find a greater anxiety significant relationship: Appukutan (14), in unemployed people and students, Kirova (9) in manual work (labourer), Nicolas (8) in farmers, manual work. The opposite result to ours where they present greater anxiety in active patients, although it is not significant. As for whether they have had previous treatment (Fig4), there are no significant differences, but it is observed that people have less anxiety when previous experiences already know that it will happen. Contrary to what was obtained by Kaako (7) where there was more anxiety when people had no previous treatment. It has been seen that it really depends on whether the previous experience was traumatic, provoking significantly greater anxiety (13). Finally, the relationship with the type of treatment (Fig. 4), there is no significant relationship, but it is observed that with aligners there is slightly more anxiety at the beginning and at six months the level of anxiety is equalized. There are no studies that relate anxiety to the type of orthodontic treatment, but we have found studies that relate anxiety to the different dental treatments and find no significant relationship between treatments invasive and non-invasive (7,12,18). Orthodontic treatment begins the moment the patient enters the dental room(Q10), they usually pass alone. It was observed that there are significant differences with patients with high anxiety presenting greater concern, but there is no scientific evidence to compare with other studies. Once they are in the dental room, their concern could be the time(Q11) and pain(Q12) they may experience sitting in the chair. The vast majority are willing to stay as long as it takes, but there is a significant relationship with patients with high levels of anxiety preferring to stay less time, Caltabiano (12) observed that the longer the appointment, the more anxious the patient feels and Muza (3) observed that 50.2% are concerned about the number of visits and the time for treatment. And even Hmud (2) observed that they were concerned about the time and the number of patients in the waiting room, thinking that they will not be treated well because of the number of patients they have waiting. While pain in the dental chair(Q12) shows a significant relationship with two variables with people with high anxiety feeling more worried about pain and with previously treated patients having less anxiety about pain. In any case, it has been seen in different studies that pain or discomfort during treatment causes high levels of anxiety (3,7,13). Once out of the dental room, the concerns are the discomfort after the check-up(Q13), which has a significant relationship with two variables, with patients with high anxiety causing greater concern and those with previous treatment showing less concern. 91-95% of orthodontic treatments cause pain (19). Pain appears within two hours of insertion and increases in the first 24-48 hours and decreases up to 7 days (19), with pain being a factor that causes increased anxiety. Meiya (21) notes that the level of anxiety increases with the onset of pain. Another discomfort that can appear with orthodontic treatment is ulcers, studies show that more ulcers appear with fixed multibracket appliances than with aligners (20-22). It is even said that these discomforts can cause changes in the quality of life. Ama Johah (23) says that the psychological impact differs a lot, some people smile more and others avoid smiling, especially with multibracket braces. Saitah alajmi (22) finds no significant difference regarding discomfort, fatigue, limitation of rest, daily sleep, daily activities, social relationships, school/work attendance between fixed multibracket appliances and aligners. However, Miller (24) significantly observed less negative impact on quality of life (functional, psychosocial, pain-related) with aligners. But it has been seen that speech modifications may occur(Q14). Our study finds a statistically significant relationship with high anxiety people and with fixed multibracket appliances at 6 months being more concerned about speech. Meiya (20) found that patients with multibracket appliances had more difficulty in pronunciation and Nedwed (19) with aligners found that 46% did not alter their speech and 93% were very confident in speaking. Contrary to Saitah alajmi (22) who found significantly more difficulties with aligners in the short term. However, Shalish (21) found no significant difference in speech between aligners and fixed appliances. In our study this is one of the main concerns at the beginning, but it disappears after 6 months and is significantly related to people with high anxiety. There is a study by Amal johah (23) where they see that a motivation for seeking treatment is to improve their dental appearance, improve facial appearance, desire to be more attractive. But the embarrassment(Q26) of wearing braces can make you decide on one treatment option or another, so that appearance is very important to include in the decision, in our study it is significantly related to people with high anxiety both at the beginning and at six months and a large decrease is observed at six months, making it one of the questions that worries them least, the same occurred in the study by María Jose González(25) who observed that the psychological impact improves with multibracket fixed appliances at 6 months after treatment. However, Maroto (26) found a significantly higher level of social and psychological and aesthetic impact after 3-6 months. Anxiety plays a key role in the effect of perceived dental impact on self-esteem in adults undergoing orthodontic treatment. Meiya (20) talks about how specifically social anxiety (fear of being rejected by peers) can occur as abnormal physical presence affects appearance, giving psychological and social consequences, making it a major cause of stress for patients, so they may have less social anxiety with aligners as they benefit from their aesthetics and may even have laughter inhibition due to aesthetics with braces (22,24). They wonder if they will be able to get used to (Q17) wearing the appliance, one of the three questions where the most and too much goes down. It is significantly related to people with high anxiety, but only at the beginning and with university students presenting greater concern about getting used to it at the beginning. Nedwed (19) in his study of aligners observed that 83% got used to them in one week, 16.7% in two weeks and 0% in 4 weeks, as they are removed for eating and brushing. Chewing problems are described during orthodontics due to tooth sensitivity and changes in occlusion and feeling particles between the teeth when distalising(19) with eating restrictions(Q18) becoming one of the major concerns at the start of treatment but being one of the lowest at 6 months, it is significantly related with patients with pre-treatment being more concerned at the start of treatment, men and with high anxiety at 6 months, and with fixed multibracket appliances at both the start and 6 months. Some studies there is no significant difference in enjoyment of food or swallowing difficulties between aligners and fixed multibracket appliances but notes that with aligners significantly better chewing ability and no restriction in amounts or types of food and with mixed appliance significantly more restriction in quantity (ate less, cut smaller pieces) and type (softer diet and avoidance of food) and more limitation in chewing (fear of throwing something away). So they eat and chew better with aligners (18,20,22). For a good evolution during treatment, collaboration and good care are necessary to avoid fractures and loss of the appliance (Q19), and this is one of the main concerns at the beginning and at 6 months. In our study there is a significant relationship at the beginning with people with high anxiety and with non-university students, with patients with aligners being more concerned at the beginning and at six months, although in Lucea’s study (27) bracket fall is the cause of the highest number of visits outside the programme for patients with fixed appliances. Márquez Rodríguez (27) sees that one dimension that worries the patient is humane treatment (dentist being in a bad mood, scolding the patient or attending to many patients at the same time) so that in our study we asked about the fear of being scolded for non-compliance(Q20) where a significant relationship was obtained with high anxiety at the beginning and at six months. In Nedweed’s study (19) patients did not show a lower degree of compliance at 3-6 months and he remarked that being well informed explains the high degree of motivation and cooperation being key in orthodontics. Orthodontic treatment involves pain, sometimes requiring the use of medication (Q21), but in our study this was one of the questions that worried them the least both at the beginning and at 6 months. There were two significant relationships at the beginning with patients who work actively and at six months with patients with high anxiety. Numerous studies have shown that the consumption of analgesics goes hand in hand with pain (21,22) and observed that the consumption of analgesics was higher in patients with multibracket appliances and less analgesics with aligners. Hmud (2) discusses a relationship between anxiety and the number of visits, explaining that anxiety increases and with a greater risk of avoidance. Orthodontic treatment requires regular (Q22) monthly visits, however, in our study it is one of the questions that at six months is of very little concern to them. It is significantly related to active patients being more worried at the beginning and at six months more worried in patients aged 25-44 years and with multibracket fixed appliances. Muza (3) showed great concern for the number of visits (50.2%) due to the fact that his sample was of low economic level, which makes them more concerned about the cost and time of treatment. However, this may not only mean one visit per month, but also due to emergencies (Q23) it may be necessary to make more visits, which could be a problem, and in our study, it is one of the six questions that at six months is of little concern to them. It is significantly related at the beginning with people with high anxiety and with multibracket fixed appliances as they usually present soft tissue lesions and descemented brackets, although generally the orthodontic urgency can be partially solved by the patient himself. Oral hygiene(Q24) is the most important in maintaining periodontal health and preventing dental caries during orthodontic treatment. If efforts to maintain good to excellent oral hygiene are unsuccessful, orthodontic treatment should be terminated. In our study it is one of the major concerns at the beginning and at six months. It is significantly related at the beginning to patients with fixed multibracket appliances being more concerned and at six months to non-university patients. Saitah alajmi (22), clearly observed better brushing and flossing with aligners, but no significant and greater plaque accumulation with afm. In fact, miethke (28) 2005 had significantly lower plaque rates with aligners and almost identical periodontal status. However, Azaripou (29) showed significantly better gingival with aligners, better periodontal health and less plaque with aligners, but not significantly. Poor hygiene and poor control during orthodontic treatment can lead to tooth loss (Q25), which is of great concern to patients both at the beginning and after six months. In his study, Jongh (30) found that 98% of patients have negative or catastrophic like loss tooth. The duration of orthodontic treatment has always been a major concern for both patients and orthodontists. it is a question that all patients ask us, but there is no established duration of orthodontic treatment as it is associated with many factors, and treatment can be lengthened (Q26). It is significantly related at the beginning and at 6 months to people with high anxiety. It is important to know the patient’s expectations because this will ultimately be the reason for the consultation. It should not be forgotten that some people seek treatment again because they do not meet expectations (23). Meiya (20) showed patients with aligners tend to have more confidence in the progress and outcome of treatment since they can see the clincheck. The fear of not meeting expectations (Q27) in our study is one of the eight questions that worries him the most at the beginning, but not after six months. Nedweed (19) who found that 89% were satisfied with aligners, although it is important to discuss in detail the succession of movements to prevent temporary dissatisfaction based on false expectations. In our study there is a significant relationship with two variables: education, with non-university patients being more worried at the beginning, and with people with high anxiety, appearing worried both at the beginning and at six months, as in the study by Hmud (2), where they observed that patients with high anxiety end up more dissatisfied. It is important to let the patient know the use retention. In our study retention(Q28) is a major concern at the beginning, decreasing at six months. It is significantly related to patients with high anxiety at the beginning. Noll colls (18) found to be one of the patients’ negative thoughts, the retention.

## Conclusions

1. The majority of patients starting orthodontic treatment have some level of anxiety, whether mild, moderate or extreme.

2. The only significant predictors are gender and occupation at six months.

3. The major concerns when starting orthodontic treatment are: Changes appearance, Eating, Loss Appliance, Hygiene, Loss Tooth, Treatment Time, Expectations and Retention.

4. The major concerns at six months are: Loss appliance, Hygiene and Loss tooth.

5. Early detection of anxiety is the best guarantee of success to avoid non-cooperation or abandonment of treatment.

This study constitutes a first phase in the analysis of the factors associated with fear and anxiety in orthodontic treatment in adults. It is necessary to introduce other scales for measuring anxiety and to study the sample at other times to see the evolution of anxiety in order to contribute to a better understanding and to develop strategies for better prevention and/or treatment.

## Figures and Tables

**Table 1:** SDAI SCALE ITEMS.

No.	SDAI Scale Item
1	I become nervous when the dentist invites me to sit down in the chair.
2	When I know the dentist is going to extract a tooth, I am already afraid in the waiting room.
3	When I think of the sound of the drilling machine on my way to the dentist, I would rather go back.
4	I want to walk out of the waiting room the momento I think the dentist will not explain what she/he is going to do in my mouth.
5	As soon as the dentist gets the needle ready for the anaesthetic, I shut my eyes tight.
6	In the waiting room, I sweat or freeze when I think os sitting down in the dentist´s chair.
7	On my way to the dentist, I get anxious at the thought that she/he will have to drill.
8	When I am sitting in the dentist´s chair not knowing what is going on in my mouth, I break into a cold sweat.
9	On my way to the dentist, the idea of being in the chair already makes me nervous.

**Table 2:** Results at the beginning after six months of treatment.

VARIABLE	BASELINE	ODDS RATIO	95% CI	P-VALUE
SEX	Female	1.8056	(0.9265, 3.6385)	0.0889
AGE	25-44	0.8077	(0.4368, 1.4820)	0.4920
EDUCATION	University	1.6923	(0.8298, 3.6093)	0.1580
OCCUPATION	Active	1.7273	(0.8143, 3.8790)	0.1665
TREATMENT TYPE	Brackets	0.6429	(0.2930, 1.3440)	0.2521
PREVIOUS TREATMENT	No	1.6941	(0.7538, 4.0987)	0.2180

VARIABLE	BASELINE	ODDS RATIO	95% CI	P-VALUE
SEX	Female	3.1481	(1.3159, 8.7859)	0.0161
AGE	25-44	0.8777	(0.4269, 1.7780)	0.7190
EDUCATION	University	1.7563	(0.7511, 4.6238)	0.2180
OCCUPATION	Active	3.1154	(1.1460, 10.942)	0.0429
TREATMENT TYPE	Brackets	1.0208	(0.4202, 2.3044)	0.9620
PREVIOUS TREATMENT	No	1.6726	(0.6431, 5.2202)	0.3262

**Table 3:** Patient responses to additional questions at baseline and six months of treatment.

Question	Time	No	A Little	Normal	A lot	Too Much
Enter the Office (Q10)	Beginning	65%	16%	13%	4%	1%
	At Six Months	75%	16%	8%	1%	0%
Pain (Q12)	Beginning	16%	21%	42%	17%	4%
	At Six Months	28%	32%	32%	6%	2%
Discomfort (Q13)	Beginning	16%	21%	45%	15%	4%
	At Six Months	30%	31%	31%	7%	1%
Speech (Q14)	Beginning	21%	23%	32%	18%	6%
	At Six Months	34%	29%	23%	11%	3%
Appearance (Q15)	Beginning	24%	19%	27%	22%	8%
	At Six Months	36%	22%	25%	13%	3%
Shame (Q16)	Beginning	40%	18%	21%	16%	5%
	At Six Months	56%	21%	17%	6%	1%
Habit (Q17)	Beginning	30%	25%	22%	20%	3%
	At Six Months	54%	26%	16%	3%	1%
Eating (Q18)	Beginning	22%	15%	32%	22%	9%
	At Six Months	37%	22%	20%	17%	3%
Loss of Appliance (Q19)	Beginning	13%	21%	22%	36%	8%
	At Six Months	22%	21%	27%	26%	4%
Being Scolden (Q20)	Beginning	31%	15%	28%	23%	3%
	At Six Months	38%	18%	30%	11%	3%
Medications (Q21)	Beginning	50%	24%	20%	5%	1%
	At Six Months	64%	17%	15%	2%	1%
Visits (Q22)	Beginning	41%	20%	33%	6%	1%
	At Six Months	45%	31%	20%	4%	0%
Emergencies (Q23)	Beginning	30%	28%	26%	12%	3%
	At Six Months	49%	25%	19%	7%	1%
Hygiene (Q24)	Beginning	17%	11%	23%	41%	8%
	At Six Months	25%	13%	25%	35%	3%
Tooth Loss (Q25)	Beginning	18%	12%	17%	31%	21%
	At Six Months	28%	13%	22%	21%	17%
Lengthening (Q26)	Beginning	16%	17%	32%	26%	9%
	At Six Months	23%	16%	34%	22%	5%
Expectations (Q27)	Beginning	13%	16%	26%	35%	11%
	At Six Months	19%	21%	32%	19%	8%
Retention (Q28)	Beginning	25%	16%	26%	24%	8%
	At Six Months	26%	21%	28%	21%	4%
		<5 Mins	>5-10 Mins	>10-20 Mins	>20-30 Mins	Any
Time (Q11)	Beginning	3%	3%	3%	8%	83%
	At Six Months	0%	3%	10%	111%	79%

**Table 4:** P-values from Fisher´s Exact Test for associations between additional questions, demographics and anxiety levels.

	Time	Age	Education	Occupation	Sex	Previous Treatment	Treatment Type	Anxiety
Enter the Office (Q10)	Beginning	0.15627	0.86108	0.73316	0.43953	0.07840	0.39240	0.00000
	At Six Months	0.10567	0.34132	0.15093	0.16299	0.36928	0.44734	0.00000
Time in the Office (Q11)	Beginning	0.45482	0.37377	0.75367	0.70307	0.48629	0.22989	0.00025
	At Six Months	0.31786	0.53177	0.31022	0.75228	0.29222	0.77804	0.08350
Pain (Q12)	Beginning	0.92433	0.92334	0.78065	0.65672	0.01529	0.62339	0.00000
	At Six Months	0.61860	0.36684	0.23171	0.38501	0.20302	0.45323	0.00096
Discomfort (Q13)	Beginning	0.75145	0.81376	0.10118	0.54069	0.02074	0.05298	0.00000
	At Six Months	0.39155	0.16489	0.16324	0.14064	0.14990	0.20282	0.00051
Speech (Q14)	Beginning	0.20199	0.21847	0.06031	0.49039	0.70996	0.24136	0.00085
	At Six Months	0.24119	0.22642	0.48814	0.16125	0.17739	0.02819	0.00754
Appearance (Q15)	Beginning	0.51608	0.77116	0.31681	0.08795	0.74763	0.73353	0.00005
	At Six Months	0.35445	0.56500	0.20190	0.78528	0.06311	0.39058	0.03977
Shame (Q16)	Beginning	0.16796	0.30410	0.29549	0.67221	0.35509	0.72689	0.01579
	At Six Months	0.75165	0.08610	0.35720	0.61182	0.51274	0.74312	0.04982
Habit (Q17)	Beginning	0.25990	0.04881	0.46806	0.28489	0.48904	0.07729	0.00031
	At Six Months	0.44356	0.09439	0.24350	0.52711	0.72987	0.12747	0.00575
Eating (Q18)	Beginning	0.50613	0.20701	0.25000	0.68824	0.04065	0.00778	0.12743
	At Six Months	0.32571	0.85773	0.50629	0.02843	0.74607	0.04755	0.02363
Loss of Appliance (Q19)	Beginning	0.54282	0.03376	0.66868	0.68304	0.57871	0.59322	0.00080
	At Six Months	0.37516	0.12826	0.35234	0.32789	0.18004	0.03568	0.97622
Being Scolden (Q20)	Beginning	0.84259	0.66648	0.22572	0.60840	0.28004	0.25113	0.01185
	At Six Months	0.86305	0.59407	0.46688	0.57328	0.52976	0.07297	0.00340
Medications (Q21)	Beginning	0.59000	0.80296	0.00620	0.24117	0.21333	0.64715	0.10023
	At Six Months	0.51017	0.46564	0.06887	0.47315	0.78046	0.37025	0.00277
Visits (Q22)	Beginning	0.33022	0.58270	0.00992	0.47279	0.72592	0.14907	0.29537
	At Six Months	0.03192	0.06909	0.11064	0.47043	0.50277	0.00667	0.07645
Emergencies (Q23)	Beginning	0.30963	0.69373	0.86616	0.52653	0.79066	0.03218	0.00017
	At Six Months	0.98381	0.53891	0.96894	0.18602	0.15425	0.69807	0.0199
Hygiene (Q24)	Beginning	0.96654	0.75912	0.21236	0.82436	0.89979	0.03288	0.8436
	At Six Months	0.17513	0.03930	0.47552	0.81810	0.43249	0.31348	0.27588
Tooth Loss (Q25)	Beginning	0.05504	0.65727	0.10767	0.97223	0.10324	0.18974	0.17487
	At Six Months	0.16239	0.05085	0.14488	0.81653	0.79717	0.80561	0.27200
Lengthening (Q26)	Beginning	0.95881	0.52523	0.23746	0.22681	0.58059	0.31236	0.00282
	At Six Months	0.73383	0.76273	0.06300	0.07896	0.41402	0.24005	0.02391
Expectations (Q27)	Beginning	0.37348	0.01110	0.28466	0.45614	0.27278	0.51365	0.00342
	At Six Months	0.38062	0.25305	0.56463	0.30496	0.72688	0.22446	0.02557
Retention (28)	Beginning	0.63535	0.81626	0.79563	0.17308	0.55806	0.76831	0.01869
	At Six Months	0.77894	0.07771	0.40259	0.52094	0.63186	0.51461	0.20117

## Data Availability

The datasets used and/or analyzed during the current study are available from the corresponding author.
